# Sexual and reproductive health knowledge of postgraduate students at the University of Cape Town, in South Africa

**DOI:** 10.1186/s12978-022-01507-6

**Published:** 2022-12-15

**Authors:** Bupe Mwamba, Pat Mayers, Jawaya Shea

**Affiliations:** 1 Department of Paediatrics and Child Health, Faculty of Health Sciences, University of Cape Town, Cape Town, South Africa; 2 Associate Professor Emeritus, Department of Health and Rehabilitation Sciences, Faculty of Health Sciences, University of Cape Town, Cape Town, South Africa; 3 School of Nursing, University of the Western Cape, Cape Town, South Africa

**Keywords:** Postgraduate students, Knowledge, Sexual health, Reproductive health, South Africa

## Abstract

**Background:**

Globally and in South Africa, university students’ knowledge of sexual and reproductive health (SRH) is low. This study was conducted in response to the dearth of information about the sexual and reproductive health knowledge of postgraduate students. Research conducted to explore the SRH knowledge of undergraduate students suggests that the level of SRH knowledge among undergraduate students is low. The aim of this study was to determine the SRH knowledge of postgraduate students with regards to contraceptives, sexually transmitted illnesses (STI), human immunodeficiency virus (HIV), Pap smear and clinical breast examination at University of Cape Town (UCT), in South Africa.

**Method:**

A cross sectional survey design was utilized, using an adapted and pretested online questionnaire. The aim of this study was to determine the SRH knowledge of postgraduate students at the UCT. Minor adjustments were made to the questionnaire to suit the South African context. Selected aspects of SRH were included in the current study: knowledge and use of contraceptives, Pap smear, clinical breast examination, STIs and HIV. These variables were considered to be general enough to be answerable by male and female respondents and are the most important considerations in reproductive health care in South Africa, as there is a high prevalence of STIs, HIV and cervical and breast cancers. All postgraduate students enrolled in the first semester of 2017 (9444) were invited to anonymously complete the online survey. Data was exported to the Statistical Package for Social Sciences (SPSS) version 23.0 and analysed using descriptive statistics such as mean, standard deviation, frequencies and percentages.

**Results:**

Four hundred and six (406) students completed the online survey, of whom 293 were female and 107 males. The age range of respondents was between 18 and 57 years, with the median age for both male and female respondents being 24 years. Six survey responses were excluded from the statistical analysis because of incomplete data. Post graduate students from the African continent comprised 90.75% of the respondents. Most respondents were white (51.50%) from both Africa and abroad. The results indicated that respondents knew about sexually transmitted infections, and human immunodeficiency virus (HIV) and acquired immune deficiency syndrome (AIDS). Female respondents were more aware of breast examination, and the role of Papanicolaou smear (Pap smear) in SRH. Almost half of the respondents in this study (49%) stated that they had no need for more information about contraceptives. Lecturers were identified as one of the top five sources of information, which suggests that the university environment provides students with important SRH-related information.

**Conclusion:**

Most postgraduate students had knowledge of sexual and reproductive health with regards to contraception, Pap smear, clinical breast examination, STIs, HIV and AIDS. Further research should focus on the relationship between SRH knowledge and usage among this population. As university lecturers were identified as an important source of information across faculties, the University should consider the incorporation of SRH education in the broader curriculum and as an integral component of student health services.

## Plain language summary

Approximately 44% of pregnancies worldwide are unplanned, with 25.1 million reported unsafe abortions, 95% of which occur in low- and middle-income countries. Youth, including university students, are particularly vulnerable to poor sexual and reproductive health (SRH) knowledge. In South Africa, university students possess low levels of SRH knowledge yet engage in high-risk sexual behaviours. The study explored postgraduate students’ knowledge of contraceptives, sexually transmitted illnesses, human immunodeficiency virus, Pap smear and clinical breast examination at the University of Cape Town in South Africa. A cross-sectional study using an online questionnaire was conducted. Descriptive statistics and multiple linear regression analysis were performed. Of the 403 respondents, 65.8% reported sexual engagement. Overall, students were knowledgeable about selected aspects of SRH and obtained their information from university academic lecturers and health professionals. In conclusion, universities have a key role in the provision of SRH information. Such information should be incorporated into the curricula and other activities, to promote healthy lifestyle choices.

## Background

Sexual and reproductive health (SRH) for adolescents and young adults is a vital component of health policy and is included in the United Nations Sustainable Development Goals [1]. Adolescents and young adults are particularly vulnerable to the consequences of poor knowledge of SRH and inadequate protection during sexual encounters. Sub-Saharan Africa, with only 12% of the global population, accounts for about 71% of the global burden of HIV infection [2]. Although there has been an increase in awareness and personal knowledge of HIV status, this is not consistent across countries [3]. Young adults (15–24 years) are also at risk of unintended pregnancies and unsafe abortions, partly due to their low levels of SRH knowledge [4]. It is estimated that 44% of pregnancies worldwide are unplanned which is attributed to low usage of contraceptives [4–6]. Of the 25.1 million unsafe abortions reported globally, 95% occur in lower-middle-income countries in Africa, Asia and Latin America [7].

Africa lags behind other continents with respect to knowledge of SRH, particularly among young people [8–10] This could be attributed to societal construction which inhibits open discussions about SRH between parents and children [11–13]. Such discussions are considered a taboo in many African families, hence many young adults learn about SRH from their peers, which could be detrimental [14, 15].

Many South African adolescents and young adults engage in unprotected sexual intercourse [16]. Richter et al. reported a median age of sexual debut for young adults as 16 years for girls and 15 years for boys with 5.8% of male respondents reporting having impregnated a girl at least once [16]. A number of studies have reported high risk-taking behaviours (multiple sexual partners and infrequent condom use) among young adults in South Africa [17, 18].

Unwanted pregnancies can negatively affect the health of individuals, families and society at large. Women have an increased risk of morbidity and mortality from abortions and the process of childbirth [14, 19]. Most health promotion programs focus mainly on adolescents below 16 years [20, 21], however unwanted pregnancy rates are higher among university students [22–24]. In Kwa-Zulu Natal Province of South Africa, 19.2% of youths were reported to have experienced unwanted pregnancies [25] and of the 326 participants, 129 (39.6%) stated that contraception was not covered in Life Orientation, which justified the lack of SRH knowledge on contraceptives among this cohort.

The majority of university students fall into the adolescent and young adult group. In South Africa, as in countries around the world, university students possess low levels of knowledge about SRH [9, 26, 27], but they are involved in high-risk behaviours [28, 29]. In a study of sexually active first year university students at a university in the Western Cape, South Africa, Abels and Blignaut reported that 62% of the respondents had not used condoms during every sexual encounter [30]. A longitudinal study (2007–2012) among students at the same university reported that 44% and 51% respectively, were sexually active prior to enrolment [31]. A survey of university students in KwaZulu-Natal, South Africa, noted that 218 of 576 students reported a vaginal sexual encounter with inconsistent contraceptive use in the previous two months [32]. A study among male students at the University of Venda, South Africa reported that the majority of the respondents had a negative attitude towards contraceptive use [33].

Existing studies in the area of SRH knowledge have mainly focused on undergraduate students, highlighting limited knowledge and low uptake of SRH services among students [25, 28, 29, 34]. Although the postgraduate student numbers in most universities in South Africa have increased over the last decade, there is limited published information on SRH knowledge among postgraduate students. The aim of the study was to determine the SRH knowledge of postgraduate students in a university in South Africa, with specific exploration of students’ knowledge and use of contraceptives, Pap smear, clinical breast examination and knowledge of STIs and HIV.

## Methods

### Study design

The aim of this study was to determine the SRH knowledge of postgraduate students with regards to contraceptives, sexually transmitted illnesses (STI), human immunodeficiency virus (HIV), Pap smear and clinical breast examination at UCT in South Africa.

A descriptive cross-sectional study was conducted, using an online questionnaire adapted from the Centre for Disease Control (CDC) reproductive health studies conducted in Albania and Malaysia on postgraduate students [35, 36]. These studies conducted separate surveys for males and females focussing on all aspects of SRH as defined by the World Health Organisation [37]. Selected aspects of SRH were included in the current study: knowledge and use of contraceptives, Pap smear, clinical breast examination, STIs and HIV. These variables were considered to be general enough to be answerable by male and female respondents and are the most important considerations in reproductive health care in South Africa, as there is a high prevalence of STIs, HIV and cervical and breast cancers [38, 39].

Data was exported to the Statistical Package for Social Sciences (SPSS) version 23.0 and analysed using descriptive statistics such as mean, standard deviation, frequencies and percentages.

### Study setting and population

The University of Cape Town, in the Western Cape Province of South Africa, was the study setting, which had 9444 registered postgraduate students in 2017 across all faculties (Faculties of Health Sciences, Commerce, Humanities, Engineering and Built Environment (EBE), Law, Science, Graduate School of Business and Centre for Higher Education development (CHED). A letter of invitation to participate in the study was emailed to all the postgraduate students in the university, with a link to the anonymous online questionnaire through the office of the student affairs. Using the formula z^2^_X_ p(1-p)/e^2^ an acceptable response rate was calculated at 403 students.

The 60-item questionnaire comprised sections dealing with socio-demographic information, SRH knowledge related to STIs, women/men’s health, contraception, pap smear, clinical breast examination, knowledge sources and prevention of HIV/AIDS. The questionnaire was in English, the language of tuition of the institution. The questionnaire was pretested online with ten undergraduate students at UCT to establish ease of access, readability, language issues and time required for completion. No modification was required.

The survey was anonymous with specific arrangements made for the IP address of the computer/mobile device to be delinked from the respondents’ submission of the questionnaire, in order to facilitate honest answers [40]. Ethical approval to conduct the study was obtained from the Human Research Ethics Committee, Faculty of Health Sciences, University of Cape Town. Permission was obtained from the Director of Student Affairs at the University to conduct the study. Information about the study, voluntary participation, risks and benefits were included in the invitation letter. Respondents who completed the questionnaire and submitted their responses were assumed to have consented to participate.

### Data management and analysis

The responses were coded, entered in Excel and data cleaned. Data was exported to the Statistical Package for Social Sciences (SPSS) version 23.0. Data were analysed using descriptive statistics such as mean, standard deviation, frequencies and percentages. Multiple linear regression analysis was performed to estimate the proportion of variance in SRH knowledge that could be accounted for by socio-demographic factors and other variables.

## Results

Four hundred and three students completed the online questionnaire. Table 1 summarises the socio-demographic characteristics of the respondents. Respondents mean age was 26.1 years. The majority were female (73.2%), single (63.3%), Christian (51.9%), white (51.6%) and pursuing honours degree (44.2%).

**Table 1:** Socio-demographic characteristics of respondents

Variable	Categories	N = 403
		N (%)
Gender		
	Male	108 (26.8)
	Female	295 (73.2)
Relationship status		
	Single	255 (63.28)
	Married	60 (14.89)
	Cohabiting	84 (20.84)
	Widowed	3 (0.74)
	Divorced	1 (0.25)
Religion		
	Agnostic	149 (36.97)
	Buddhist	1 (0.25)
	Christian	209 (51.89)
	Hindu	12 (2.98)
	Muslim	9 (2.23)
	Orthodox	3 (0.74)
Race		
	Asian	2 (0.5)
	Black African	117 (29.03)
	Coloured	37 (9.18)
	Indian	28 (6.95)
	White	208 (51.6)
	Others	11 (2.73)
Educational level		
	Post-Graduate Diploma	67 (16.67)
	Honours degree	178 (44.17)
	Masters’ degree	113 (28.04)
	Doctor of Philosophy	31 (7.69)
	Postdoctoral degree	14 (3.47)
Faculty		
	CHED	2 (0.5)
	Commerce	70 (17.37)
	EBE	54 (13.4)
	Graduate School of Business	7 (1.74)
	Health	90 (22.23)
	Humanities	112 (27.79)
	Law	20 (4.96)
	Science	48 (11.91)

*CHED*  Centre for Higher Education Development; *EBE* Engineering and Built Environment

### General knowledge of SRH

Two hundred and sixty-five (65.8%) respondents indicated they were currently in a sexual relationship. The majority (90.4%) reported knowledge about the menstrual cycle and a woman’s fertility period. 89.4% of respondents agreed that women had the right to make personal decisions about pregnancy and termination.

### Knowledge of contraceptive methods

**Fig. 1:**
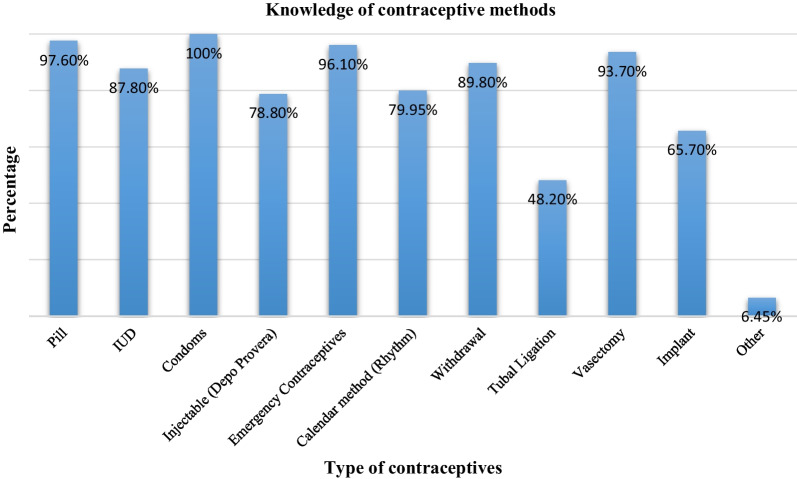
Knowledge of contraceptive methods

Respondents indicated that they had good knowledge of contraceptive methods (Fig. 1). 92.5% reported knowing how to use their chosen method and 87.8% had previously used their chosen method. The contraceptive methods most utilised were condoms (87.1%), oral contraceptives (68%) and the withdrawal method (36%).

### Knowledge of STI symptoms

Most male respondents knew that burning pain on urination (98.01%), redness in the genital area (97.02%), genital sores (97.77%), genital itching (96.53%) and discharge from the penis (95.78%) were symptoms of STIs in men (Table 2). Similar responses were reported for female students. Symptoms about which all respondents were less knowledgeable were abdominal pain (60.3%), weight loss (56.33%) and difficulty in impregnation (47.89%).

**Table 2:** Knowledge of STI symptoms by gender

STI symptoms	Male		Female		
	Yes	No	Yes	No	Don’t know N (%)
	N (%)	N (%)	N (%)	N (%)	
Abdominal pain	243 (60.3)	160 (39.7)	296 (73.45)	39 (9.68)	68 (16.87)
Discharge from the penis	386 (95.78)	17 (4.22)	371 (92.06)	18 (4.47)	14 (3.47)
Foul smelling discharge	368 (91.32)	35 (8.68)	388 (96.28)	1 (0.99)	14 (3.47)
Burning pain on urination	395 (98.01)	8 (1.99)	385 (95.53)	4 (0.99)	14 (3.47)
Redness/inflammation in the genital area	391 (97.02)	12 (2.98)	390 (96.77)	1 (0.25)	12 (2.98)
Swelling in genital area	382 (94.79)	21 (5.21)	368 (91.32)	10 (2.48)	25 (6.20)
Genital sores/ulcers or warts	394 (97.77)	9 (2.33)	390 (96.77)	2 (0.5)	11 (2.73)
Genital itching	389 (96.53)	14 (3.47)	381 (94.54)	4 (0.99)	18 (4.47)
Weight loss	227 (56.33)	176 (43.67)	218 (54.09)	88 (21.84)	97 (24.07)
Hard to get a woman pregnant	193 (47.89)	210 (52.11)	194 (48.14)	123 (30.52)	86 (21.34)
Refuse to answer	–	–	29 (7.20)	206 (51.12)	168 (41.68)

### Knowledge of HIV transmission and prevention

Table 3 summarises respondents’ knowledge of HIV transmission. All respondents knew about heterosexual transmission of HIV and most were aware of HIV transmission through unsterile syringes and needles (98.3%) and unprotected male-to-male sexual intercourse (97.8%); however, there was a greater spread of reported knowledge about other modes of transmission. All respondents reported good knowledge of HIV risk reduction mechanisms such as use of condoms, abstinence, requesting that a partner be tested, use of sterile needles/syringes and not sharing razors and other personal equipment.

**Table 3:** Knowledge of HIV transmission

Mode of transmission	Yes	No	Don’t know
	N (%)	N (%)	N (%)
Blood transfusion	395 (98.0)	8 (2)	0 (0)
Using public toilets	6 (1.5)	380 (94.3)	17 (4.2)
Kissing	67 (16.6)	332 (82.4)	04 (1)
Unprotected sex between man and woman	403 (100)	0 (0)	0 (0)
Unprotected sex between men	394 (97.8)	3 (0.7)	6 (1.5)
Shaking hands	0 (0)	403 (100)	0 (0)
Using non-sterile syringes/needles	396 (98.3)	7 (1.7)	0 (0)
Mosquito bites	26 (6.5)	350 (86.8)	27 (6.7)
Sharing plates/forks/glasses with someone with HIV or AIDS	6 (1.5)	387 (96.0)	10 (2.5)
Mother with HIV to her baby during pregnancy or delivery	373 (92.6)	23 (5.7)	7 (1.7)
Mother to child through breast milk	271 (67.2)	74 (18.4)	58 (14.4)
Manicure, pedicure or haircut	28 (6.9)	359 (89.1)	16 (4.0)
Dental or surgical treatment	156 (38.7)	202 (50.1)	45 (11.2)

### Knowledge of pap smear and clinical breast examination

Female respondents were knowledgeable about the Pap smear (68.49%) and clinical breast examination (67.99%). Within the male respondents (N = 108) 77.78% and 86.11% respectively, were knowledgeable about pap smears and clinical breast examination.

### Sources of knowledge about STIs, HIV and contraception

Respondents reported that they obtained information about STIs and HIV from lecturers (23.82%) and the Internet (22.83%). For information about contraceptives, the primary source (28.61%) were health professionals such as doctors, nurses, midwives and pharmacists. Also, less than one-third (21.39%) obtained information from the Internet. Family members were least likely to be consulted for information on STIs, HIV (5.71%) and contraception (7.21%).

### Determinants of SRH

Multiple linear regression analysis (Table 4) revealed that knowledge about the Pap smear was positively affected by age and negatively affected by gender; thus indicating that older age was associated with increased knowledge of Pap smear. Male students reported less knowledge that females about the Pap smear. Knowledge about clinical breast examination was associated with age, gender, faculty, religion and race. Being female, white race, a Health Sciences student and of the Christian faith was associated with increased the knowledge of breast examination. Gender was the only factor found to be associated with contraceptive knowledge whereas knowledge about STIs and HIV was associated with race and age.

**Table 4:** Determinants of SRH (multiple regression analysis)

Variable	Pap smear		Clinical breast exam		Contraceptives		STIs/HIV and AIDS	
	Coef.	Std. error	Coef.	Std. error	Coef.	Std. error	Coef.	Std. error
Relationship status	0.0095	0.1290	0.0061	0.0120	0.0032	0.0037	0.0091	0.01086
Age	0.0064*	0.0024	0.0046**	0.0022	− 0.0004	0.0007	0.0037*	0.0020
Gender	− 0.1688*	0.0345	− 0.0837*	0.0321	− 0.2833*	0.0099	− 0.0329	0.0289
Education	− 0.0032	0.0104	− 0.0044	0.0097	− 0.0009	0.003	− 0.0081	0.0088
Faculty	− 0.0022	0.0079	− 0.012***	0.0073	− 0.0019	0.0023	− 0.0047	0.0066
Religion	− 0.0083	0.0025	− 0.0054*	0.0023	− 0.0008	0.0007	–	–
Race	0.0179	0.0115	0.0429*	0.0107	0.0020	0.0033	0.0210*	0.0093
Constant	1.9500*	0.1330	1.8179*	0.1240	1.0413*	0.0328	2.8083*	0.0918

^*^Sig.1%: **Sig.5%: ***Sig.10%

A similar pattern was seen in students’ responses with regards to symptoms that may be seen in a female with an STI. Non-Specific symptoms response had less than 90% in comparison with specific symptoms which scored above 90%, signifying high knowledge of STIs among postgraduate students.

## Discussion

The study investigated postgraduate students’ knowledge of specific aspects of reproductive health. 65.8% of respondents were in a sexual relationship at the time of the study. Postgraduate students were overall knowledgeable about sexual and reproductive health, in particular contraceptive use, especially condoms, symptoms associated with STIs and transmission of and prevention of HIV.

### General knowledge about sexual and reproductive health

Many respondents were knowledgeable about the menstrual cycle because they knew the time point at which a woman would get pregnant. This cannot be generalized because most respondents were female. Females are aware of the menstrual cycle which begins at the onset of menarche and ends at menopause. However, respondents had limited knowledge on lactational amenorrhea as a contraceptive method, which could be attributed to the lack of promotion of this method as it requires some level of commitment to achieve its contraceptive effect. Also reported similar findings to those found in this study with regards to the contraceptive effect of breast feeding [36, 41, 42]. However, it is important to educate people about available contraceptives including natural methods such lactational amenorrhea. The findings in this study differ from a Malaysian study, which reported that postgraduate students’ knowledge of sexual and reproductive health was unsatisfactory [36]. In the current study, many respondents also agreed that a woman has the right to SRH, which includes the woman’s right to pregnancy, including either to have an abortion or not. This shows that the current young adults have information on SRH rights. Sexual and reproductive health rights of women is a human rights priority worldwide [43], and in South Africa this is enshrined in the Constitution [44]. The WHO have emphasised the need for women to have control over their sexual and productive health needs to help meet targets of the sustainable development goals on health [45].

### Knowledge of contraceptives and symptoms of STIs

Respondents’ were knowledgeable about contraceptives, especially with regards to condoms (100%) and oral contraceptives (97.6%). The finding is in line with other studies in KwaZulu-Natal (South Africa), Kenya and Tanzania were research participants were knowledgeable about contraceptives [46–48]. Respondents were also knowledgeable about the symptoms of STIs. Such knowledge is a facilitator of healthy sexual and reproductive behaviour and may promote early health seeking behaviour for early diagnosis and treatment/management of symptoms [49, 50].

### Knowledge of transmission and prevention of HIV

Nearly all the respondents reported knowledge of modes of HIV transmission and preventive measures. Similar findings were reported in Ghana, United Arab Emirates and Cameroon were respondents had knowledge of modes of HIV transmission and preventive measures [51–53]. Knowledge of HIV transmission and prevention is key to mitigating the disease. In a country with a high HIV burden, (7.52 million people living with the disease [38], knowledge, however, may not be enough to reduce the rate of new infections and promote safe practice. Prevention strategies that integrate biomedical, behavioural, social and structural prevention interventions are required to effectively reduce HIV risk-related behaviours, especially in young people [54].

### Knowledge of Pap smear and clinical breast examination

The study found female respondents were more knowledgeable of Pap smear (68.49%) and clinical breast examination (67.99%) compared to their male counterparts which was highlighted in the regression analysis. A study with female students in India reported that majority (97.96%) of the respondents had no knowledge of pap smear [55]. Cervical and breast cancers are the leading cause of morbidity and mortality in women who are of reproductive age [56] and thus the knowledge reported in the current study has positive implications for health and prevention of illness. Since females were more knowledgeable of Pap smear and clinical breast examination, it is essential to bridge the knowledge gap for men to allow them to play the required supportive roles for women.

### Respondents’ sources of information

Majority of the postgraduate students reported sourcing information about STIs and HIV from lecturers and that of contraception information from health professionals. The finding could be attributed to the significant role lecturers and health professionals play in health promotional activities especially on SRH [57]. It is suggested especially for the university to maintain this endeavour. However, the study shows family and friends playing a lesser role in the provision of information. This may indicate the cultural, religious and other taboos which limit open discussion of SRH matters between parents and their children [58–61]. In South Africa, families who openly discuss SRH issues with their children are in the minority [60]. This is an area for further study.

### Implications and recommendations

Given the findings, university policy makers and health services should ensure the incorporation of SRH education in the curriculum across faculties, as most of the respondents listed lecturers as their source of information on SRH. This will be particularly important for males, since females were identified to be more knowledgeable compared to males. Bridging the knowledge gap for males would allow them to play their required roles, as men are reported to have control over financial resources in households [62, 63]. In addition, the findings demonstrated that families play a lesser role as source of information on SRH to young adults. Therefore, it is suggested that interventions be put in place to equip parents with skills on how to communicate with young adults on SRH issues. Evidence demonstrate that parents lack the capacity to communicate on SRH [60]. Equipping the young adults with knowledge on SRH would promote good health outcomes for them [61]. Lastly, it is recommended for studies to be carried out on the relationship between SRH knowledge and access to SRH services. This will help determine whether knowledge of SRH issues translate to access and utilisation of SRH services.

## Conclusion

The study demonstrated that postgraduate students were knowledgeable about SRH issues comprising of contraception, STIs, HIV and AIDS, Pap smear and clinical breast examination. Lecturers and health professionals were reported to be the main source of information. It is recommended for decision makers and the university to incorporate SRH issues into the curriculum. This will allow the university to play a holistic developmental role in preparing postgraduate students for a healthy lifestyle.

## Data Availability

The dataset is available on request from the corresponding author.

## Abbreviations

ADM: Advanced midwife
AIDS: Acquired immune deficiency syndrome
CDC: Centers for Disease Control and Prevention
DISCHO: Discrimination and Harassment Office (UCT)
HIV: Human immunodeficiency virus
HPV: Human papilloma virus
FHSHREC: Faculty of Health Sciences Human Research Ethics Committee
MCH: Maternal and child health
MPhil: Master of Philosophy in Maternal and Child Health
NASRHRFS: National Adolescent Sexual and Reproductive Health and Rights Framework Strategy
NN: Neonatal nurse
Pap smear: Papanicolaou smear
RH: Reproductive health
SA: South Africa
SRH: Sexual and Reproductive Health
STI: Sexually transmitted infections
SSA: SubSaharan Africa
UCT: University of Cape Town
UKZN: University of KwaZulu Natal
UNAIDS: United Nations Program on HIV and AIDS
UNFPA: United Nations Population Fund
WC: Western Cape Province
WHO: World Health Organization
WMA: World Medical Association
